# Successful treatment of a patient with small cell lung cancer receiving hemodialysis, with concurrent oral etoposide and radiotherapy: A case report

**DOI:** 10.1097/MD.0000000000022637

**Published:** 2020-10-02

**Authors:** Feng Gao, Xiaofeng Cong, Ziling Liu

**Affiliations:** Deparment of Oncology, First Affiliated Hospital, Jilin University, Changchun, Jilin, PR China.

**Keywords:** case report, etoposide, hemodialysis, small cell lung cancer

## Abstract

**Introduction:**

Small cell lung cancer (SCLC) is an aggressive malignancy that progresses rapidly and easily relapses. To the best of our knowledge, advances have been minimal for decades and the first-line treatment is still platinum-etoposide and radiotherapy. However, elderly patients with severe renal failure who suffer from SCLC usually show more serious drug-related side effects. A large proportion of them cannot tolerate the standard treatment, and their prognosis is poorer compared with that of younger patients. Presently, oral etoposide capsules may be accepted as a replaceable option. We report the case of a male patient with SCLC on hemodialysis who was successfully treated with concurrent oral etoposide monotherapy and radiotherapy and achieved excellent outcomes.

**Patient's Concerns:**

A 63-year-old man with severe renal failure was diagnosed with SCLC.

**Primary Diagnoses:**

SCLC was diagnosed using transbronchial biopsy.

**Interventions:**

He received concomitant single-agent oral etoposide (6 cycles) and local radiotherapy. Etoposide 100 mg once daily combined with thoracic radiation treatment (2 Gy/f, total DT: 50 Gy/25 f), was subsequently followed by prophylactic cranial irradiation plus anlotinib.

**Outcomes:**

The patient achieved complete response after 1 cycle and the subsequent treatment was effective without any kidney damage and other severe side effects.

**Conclusion:**

Though etoposide capsule is an old drug, its use should be considered in SCLC patients with renal insufficiency undergoing hemodialysis. However, treatment guidelines and research data for such patients are still lacking and further studies are needed. Although recent research focuses mainly on new drugs, some old drugs like etoposide which can bring unexpected positive effects should not be neglected.

## 1 Introduction

Although the relationship between chronic kidney disease (CKD) or hemodialysis and the high risk of lung cancer has not been fully documented, existing studies show that there is an increasing tumor prevalence in hemodialyzed patients.[^1^,^2^] This is probably associated with the susceptibility of infection, weakened immunity, and altered DNA repair mechanisms.^[3]^ Patients with cancer and CKD on hemodialysis have a limited drug choice; therefore, the fewer therapeutic options available to them is a tricky problem. It is more intractable in patients with concomitant renal failure and small cell lung cancer (SCLC). To the best of our knowledge, lung cancer is the most leading cause of cancer-related deaths and is the most incident carcinoma worldwide. Compared with non-small cell lung cancer (NSCLC), the rate of SCLC research is slower and this has frustrated oncologists globally- there has been no significant clinical breakthroughs for at least 20 years.^[4]^ Presently, platinum-etoposide chemotherapy is the gold standard chemotherapy regimen. Prophylactic cranial irradiation (PCI) is also recommended to prevent brain metastases. However, the side-effect and toxicity may limit the tolerance and curative effect in elderly patients and those with a poor performance status. Particularly, platinum worsens renal impairment, but oral etoposide produces good outcomes in treating concomitant SCLC and end-stage renal disease (ESRD) with slight adverse effects. Herein, we report the case of an elderly SCLC patient with severe renal failure successfully treated with oral etoposide monotherapy as first-line treatment combined with local radiotherapy.

## 2 Patient information

A 63-year-old male with a smoking history of 30 pack-year was found to have a significant creatinine elevation (>600 μmol/L) in April 2018 when he came for a routine check-up. With a diabetes history of more than 20 years and a poor glycemia control, it was diagnosed as diabetic nephropathy. Since then, he has been on regular hemodialysis in the local hospital.

## 3 Clinical findings

In February 2019, he was admitted in our hospital for aggravating cough and exertional dyspnea. Chest computed tomography (CT) scans revealed a right hilar mass with unclear edges extending to the right middle lobe and mediastinum accompanied by enlarged bilateral hilar and mediastinal lymph nodes with a mild right pleural effusion. Transbronchial biopsies were performed and the pathology conformed to SCLC.

His vital signs were normal and no obvious abnormalities were found in his physical examination. His Eastern Cooperative Oncology Group Performance Status (ECOG-PS) score was 1. He had no other past medical history such as hypertension, heart disease or liver dysfunction apart from diabetes.

## 4 Timeline

Figure 1.

**Figure 1:**
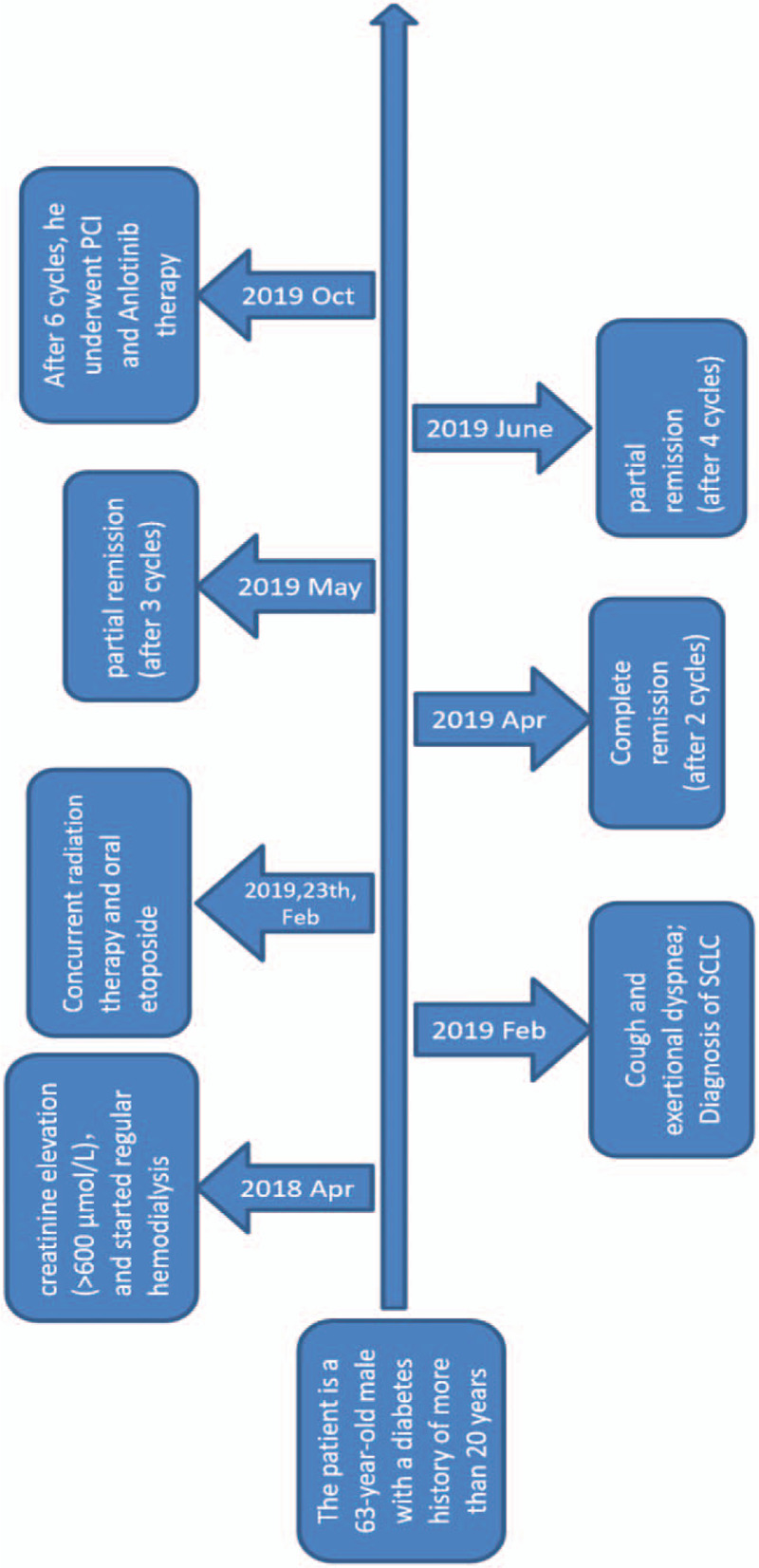
Timeline.

## 5 Diagnostic assessment

At the time of admission in February 2019, the laboratory examinations revealed a creatinine level of 391.5 μmol/L with positive urinary protein. The glomerular filtration rate (GFR) was 14.4 ml/minutes accompanied by decreased urine output, which was considered as ESRD.^[5]^ His white blood cell count was normal, hemoglobin was 105 g/L, and the platelet count was 259 x 10^9^/L. The international normalized ratio (INR) was 1.10, fibrinogen level was 6.30 g/L, D-dimer value was 283 μg/L, and PRO-LPBN level was 2750 pg/ml (normal range 0–125 pg/ml). Serum chemistry examination results showed: potassium, 3.53 mmol/L; sodium, 134.9 mmol/L; and chloride, 98.8 mmol/L. The level of cytokeratin-19 fragment (CYFRA21-1), neuron-specific enolase (NSE), carcinoembryonic antigen (CEA) and some other cancer biomarkers were within normal range. Further examination did not reveal any other metastases. The tumor, based on the overall results above, was assessed as a limited-stage SCLC. Given that he was an elderly male with chronic renal failure, fluid restriction was taken into consideration to prevent volume overload.

## 6 Therapeutic intervention

Thus, we administered concurrent chemoradiotherapy with oral etoposide 100 mg/day on day 1 to 14 every 21 days, plus thoracic radiotherapy (image-guided radiation therapy [IGRT]; 2 Gy/f, total DT: 50 Gy/25 f). Meanwhile, the patient was undergoing his routine hemodialysis as before.

## 7 Follow-up and outcomes

**Figure 2:**
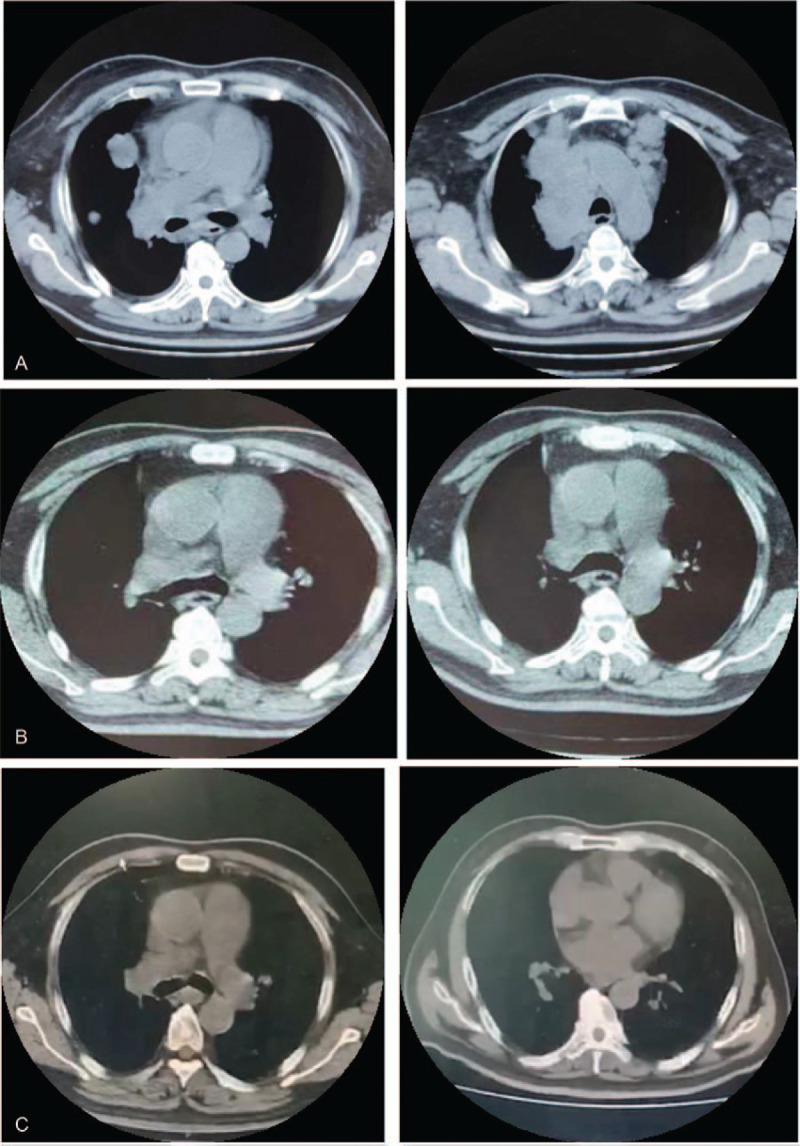
A. CT scan showed right hilar mass with unclear edges extending to the right middle lobe and mediastinum accompanied by a few right pleural effusion. B. Two cycles after treatment (May 2, 2019), therapeutic evaluation was considered as a complete remission (CR) and the size of mass reduced remarkably. C. Four cycles after treatment (June 20, 2019), the efficacy was partial remission (PR).

After 2 treatment cycles, the patient achieved a favorable radiological response. The chest CT scan showed that the extent of the lesion had reduced dramatically or disappeared partially, the enlarged lymph nodes became obviously smaller, and the right pleural effusion decreased. Therapeutic efficacy was considered as complete remission (CR) according to the Response Evaluation Criteria in Solid Tumors (RECIST) version 1.1. (Fig. 2A). After the third chemoradiotherapy cycle, there was a remarkable tumor reduction observed radiologically, and pleural effusion almost completely disappeared. This was considered as partial remission (PR, Fig. 2B). Four cycles later, a CT scan was conducted and a further lung mass reduction was observed, that is, another PR (Fig. 2C). The chemoradiotherapy was continued until a total of 6 cycles were completed and the disease remained stable. During the whole treatment process, the patient developed slight myelosuppression which was controllable and tolerable and no other significant adverse events. After that, he underwent PCI and was put on anlotinib therapy based on the ALTER 1202 trial and CSCO Lung Cancer Practice Guidelines (version 2019) to prolong progression-free survival (PFS) and avoid recurrence. For personal reasons, the patient did not provide his consent for further regular follow-ups.

## 8 Discussion

SCLC, which is designated as “recalcitrant cancer,”^[5]^ is a lethal disease representing approximately 15% of all lung cancer cases with a 5-year survival rate of only 19.8%.^[6]^ SCLC is characterized by its high aggressiveness and malignancy, early metastatic dissemination, rapid growth, and easy to recur, leading to a less chance of surgery. Chemoradiotherapy can effectively treat patients with limited-stage disease. Although those with extensive-stage disease will have a remarkable initial response, they will relapse and manifest with clinical drug resistance and a dismal prognosis. Over the last decades, the medical community has witnessed the accelerating pace of molecular-targeted treatment and immune checkpoint inhibitors in NSCLC, in contrast, the counterpart in SCLC is a little cloudier. Molecular-targeted therapy and immunotherapy for lung cancer are in full swing. Research focused on the genomic landscape of SCLC and a molecularly-driven approach such as Bcl-2 protein and the key node DLL3 have been included.^[7]^ Moreover, Deneka et al presented an emerging novel approach – drug conjugates, aimed to concentrate active components within the tumor tissue to ensure full antitumor activity.[^8^,^9^] It was shown that antiangiogenic molecules (e.g., bevacizumab and sorafenib) used in first-line or maintenance therapy of SCLC cannot improve survival.[^10^,^11^,^12^] However, apatinib, an antiangiogenic drug has a confirmed favorable efficacy in treating refractory or relapsed SCLC in some trials and cases.[^13^,^14^] Furthermore, the use of anlotinib (a new type of small molecule and multi-target tyrosine kinase inhibitor which has the effect of anti-tumor angiogenesis and tumor growth) in third-line and further-line therapy in SCLC in the ALTER 1202 (NCT03059797) trial has promising outcomes. In view of excellent outcomes in the ALTER 1202 trial, the use of anlotinib as third-line and further-line therapy or maintenance therapy to prolong PFS was approved by the CSCO in China to treat SCLC.^[15]^ However, results of the use of immune checkpoint inhibitors in SCLC seem disappointing. Nevertheless, research is ongoing. For SCLC (inoperable) patients, first-line treatment is still based on platinum-etoposide/irinotecan, and topotecan serves as a selectable drug during tumor relapse.

Platinum drugs play a key role in SCLC treatment, in which cisplatin and carboplatin are the most common. By formation of DNA crosslinks, cisplatin induces DNA damage and causes apoptosis in cells while causing several well-known toxicities (particularly kidney damage).^[16]^ The key approach to its clearance or excretion lies within glomerular filtration and tubular secretion, with a certain degree of cisplatin accumulation in the kidney.[^17^,^18^] Although large-dose infusion can reduce renal toxicity, cisplatin is not recommended when GFR is less than 30 ml/minutes for those patients with pre-existing renal failure.^[19]^ Carboplatin has a similar structure and mechanism of action as cisplatin.^[20]^ Compared with cisplatin, carboplatin has a lower excretion and longer half-life, which gives rise to longer lasting effects.^[21]^ Several previous studies have shown no significant difference in the efficacy of cisplatin and carboplatin as first-line therapy in SCLC, but the former shows more non-hematological toxicities (such as renal toxicity) whereas the latter exhibits higher rates of myelotoxicities.^[22]^ Hence, carboplatin causes less renal damage, but pre-existing renal failure will increase its plasma level, contributing to other systemic dysfunctions.^[20]^

Etoposide (VP-16) is a semi-synthetic derivative of podophyllotoxin and a cell cycle-specific cytotoxic drug, whose major mechanism is interaction with topoisomerase II or forming free radicals to destroy DNA to exert its anti-tumor activity.^[23]^ It had been established as the standard treatment drug for SCLC decades ago and is also widely used in clinical diseases such as breast cancer, leukemia, lymphoma, ovarian tumor, bladder cancer, etc. Even though its use could be suppressed by the promising results of various novel drugs, it cannot be ignored by clinicians given that is as an old but classic antitumor tool for SCLC. For intravenous etoposide, considering that its clearance is highly related to creatinine clearance and renal function, a dose adjustment and administration schedule based on individual characteristics have been recommended.[^24^,^25^]

The high sensitivity and good response of SCLC to platinum-etoposide chemotherapy are proven. However, SCLC individuals, when also undergoing hemodialysis, are often prone to be undertreated. The therapeutic drug choices are limited due to renal insufficiency, and they often tend to be excluded by various clinical trials, leading to the lack of relevant data and sufficient clinical evidence in such groups of patients. Authoritative treatment guidelines have not been clearly established so far. Hence, a rather high proportion of SCLC patients who also suffer renal failure cannot be managed with an optimum therapy and appropriate dose.

Some studies have indicated that it is feasible and effective to use standard dose chemotherapy of platinum-etoposide for SCLC patients undergoing hemodialysis.[^26^,^27^,^28^] Watanabe et al earlier elucidated the feasibility of standard-dose administration of combination chemotherapy involving cisplatin and etoposide with tolerable and manageable toxicities via a dose escalation trial.^[26]^ Subsequently, Inoue et al confirmed that the carboplatin and etoposide scheme can be administered with a relatively high-dose in SCLC patients with renal failure. Takezawa et al also suggested that carboplatin-etoposide chemotherapy plus hemodialysis led to a drug concentration within an effective range.[^28^,^29^] However, their study involved a very small sample size and therefore, studies with larger sample sizes are required. Regarding targeted therapy and immunotherapy in treating hemodialysis patients with lung cancer, several cases have been reported, but none involved SCLC patients.[^30^,^31^,^32^] Togashi et al described a case that developed SCLC during hemodialysis, and achieved long-term survival after multiple chemotherapy courses.^[33]^ Moreover, a 49-year-old hemodialyzed man with limited-stage SCLC successfully treated with standard-dose irinotecan was reported in Korea.^[34]^ In addition, several domestic and international literatures have reported that etoposide alone was used as second-line or maintenance treatment in advanced SCLC. Few papers of oral etoposide as first-line treatment in elderly SCLC patients with comorbidities or poor performance status in the 1990s have shown the superiority of oral etoposide.[^35^,^36^,^37^] However, the cases of oral etoposide capsule in patients with SCLC undergoing dialysis have not yet been reported.

Based on the need to be prudent, our patients specific situation, the key role and good initial efficacy of etoposide in SCLC, and the need to lighten the kidney load, we adopted the regimen composed of oral etoposide and local radiotherapy as first line. Differ from intravenous application, the pharmacokinetics of oral etoposide are imprecise as a result of considerable inter- and intra-patient biovariability. Therefore, we can consider that with the same dose of etoposide capsule, some patients may develop unexpected serious toxic reactions, whereas others will not attain full therapeutic effect.^[38]^ The dosage of etoposide usually depends on the body surface area. Myelosuppression is the main toxicity reaction of oral etoposide. Elderly patients experience more severe toxicity compared to young patients with equivalent drug exposure.^[39]^ Our patient did not develop serious side effects during treatment.

Toffoli et al indicated that a lower dose (50–100 mg/day, for 14–21 days) of etoposide capsule would produce better treatment results; whereas, etoposide absorption reaches saturation levels at a dose of more than 200 mg/day. The antineoplastic effect of etoposide is schedule-dependent and prolonged exposure could improve its antitumor activity. Considering the effect of etoposide clearance on the kidney function, it is necessary to adjust the dose according to the creatinine clearance rate to correct the exposure of the drug in patients with renal insufficiency. However, it is difficult to optimize the dosage of oral etoposide because of its bioavailability and pharmacokinetic variability; this is one of its drawbacks.[^39^,^40^] Oral etoposide has enormous advantages in improving the quality of life and economic benefits of cancer patients. Previous studies found that many patients prefer oral medication rather than intravenous infusion.^[41]^ Oral drug administration is advantageous in that, peripheral venule injury caused by the direct infusion of toxic chemicals can be avoided, there is no need to worry about venous damage or other related complications from the infusion needle, venous indwelling needle, or peripherally inserted central catheter (PICC). Nevertheless, thoracic radiotherapy in SCLC is of significant importance and should be started as early as possible.

As a special group of tumor patients, the treatment of elderly patients with renal failure will be remarkably influenced. In our case, a single-agent etoposide capsule combined with radiotherapy effectively and safely treated an elderly SCLC patient undergoing hemodialysis. However, we did not perform pharmacodynamic and pharmacokinetic monitoring or collect individual bioavailability data in the course of therapy. Thus, there was no contribution to individual dosage analysis based on the variability in the bioavailability of etoposide. A couple of studies have explored the bioavailability and pharmacokinetic variability of oral etoposide by establishing research models and tried to adjust the dosage according to individual characteristics to achieve an optimal treatment effect.[^39^,^40^] Further studies on optimizing the application scheme of oral etoposide as well as authoritative treatment guidelines for hemodialysis patients with SCLC are needed. Our report suggests that, though the single oral etoposide formulation cannot be recommended as a first-line therapy, it is suitable for patients who are ineligible for chemotherapy.

## 9 Patient perspective

Before coming to hospital, I had a lot of difficulties during breathing, especially after exercise, but after receiving treatment here, I felt so relieved and much better than before.

## Acknowledgments

The authors would like to thank the patient and his family for their agreement on the publication of this report.

## Author contributions

**Writing – original draft:** Xiaofeng Cong.

**Writing – review & editing:** Ziling Liu.
